# The Low Dose of *Saccharomyces cerevisiae* Is Beneficial for Rumen Fermentation (Both In Vivo and In Vitro) and the Growth Performance of Heat-Stressed Goats

**DOI:** 10.3390/microorganisms10101877

**Published:** 2022-09-20

**Authors:** Ligang Xue, Shuyi Zhou, Dan Wang, Fangyu Zhang, Junfeng Li, Liyuan Cai

**Affiliations:** 1 College of Animal Science and Technology, Jilin Agricultural Science and Technology University, 77 Hanlin Road, Jilin 132109, China; 2 Hainan Provincial Animal Husbandry Technology Promotion Station, Haikou 570100, China; 3 College of Veterinary Medicine, Jilin Agricultural University, Changchun 130118, China; 4 Institute of Animal Husbandry and Veterinary Medicine, Jilin Academy of Agricultural Sciences, Shengtai Street, Changchun 130033, China; 5 Department of Animal Nutrition and Feed Science, College of Animal Science and Technology, Huazhong Agricultural University, Wuhan 430070, China

**Keywords:** goats, heat stress, *Saccharomyces cerevisiae*, rumen fermentation, growth performance

## Abstract

This study aimed to investigate the effects of *Saccharomyces cerevisiae* on rumen fermentation and the growth performance of heat-stressed goats. The fermentation experiment was conducted using *Saccharomyces cerevisiae* added at 0‰ (HS1), 0.30‰ (SC1), 0.60‰ (SC2), and 1.20‰ (SC3) of the dry matter (DM) weight of the basal diet. The results showed that supplementing with 0.60‰ (SC2) could increase the pH, acetic acid to propionic acid ratio, the concentrations of ammonia nitrogen, total volatile fatty acids, acetic acid, propionic acid, butyric acid, and the degradability of DM, neutral detergent fiber, and acid detergent fiber in rumen fluids of heat-stressed goats. In the feeding experiment, twelve heat-stressed goats were assigned to a 4 × 4 Latin square experimental design, and the *Saccharomyces cerevisiae* supplement levels are similar to the fermentation experiment above. Similar effects on rumen fermentation and digestibility parameters were obtained with a supplement with 0.60‰ of *Saccharomyces cerevisiae* (SC2A) compared to the fermentation trial. Moreover, in the SC2A group, the DM intake and average daily gain also increased significantly compared with other groups. These results suggested that a low dose of *Saccharomyces cerevisiae* can still effectively improve the rumen fermentation and growth performance of heat-stressed goats.

## 1. Introduction

In the past decade, the development of intensive goat production in China has increased at a rapid pace, especially in the Jianghuai region (the longitude range from 28° N to 34° N and 114° E to 121° E) [1]. Goats are suitable for breeding in a cool and dry environment [2]. However, the climate of this region is characterized by high environmental temperature and relative humidity in summer [1]. Therefore, goats in this region are easily exposed to heat stress during the summer [3]. Heat stress could lead to several adverse influences on rumen fermentation, including decreased ruminal pH values, the concentration of ruminal volatile fatty acids (VFAs), the activities of digestive enzymes, and the feed digestibilities [3,4,5]. These adverse influences eventually cause a significant decline in growth performance, and then lead to economic loss [6].

Probiotics are live microbial additives which are administered at an appropriate dose and benefit the host [7]. Probiotics are widely used in the production of ruminants because they have the advantages of non-toxic side effects, no resistance, and no residue and improve feed digestion, production performance, and health status [8,9]. Yeast is an important group of probiotics commonly used in ruminant production and nutritional studies [8]. In the rumen, it could consume oxygen to maintain an oxygen-free environment and boost the abundance of rumen anaerobic bacteria, then promote rumen fermentation [10,11]. *Saccharomyces cerevisiae* can promote the growth of lactate utilizing bacteria, improve the utilization capacity of lactate, and then stabilize the ruminal pH value [12,13]. Previous studies reported that the combined high-starch diet with *Saccharomyces cerevisiae* cultures could improve the digestibilities of fiber and starch and reduce the risk of subacute rumen acidosis in dairy cattle [14]. Previous studies found that yeast supplementation significantly decreased the concentration of NH_3_-N in the rumen of goats, cattle, and bulls [11,15,16]. Dietary supplementation with yeast induces a significant increase in the concentration of TVFA in the rumen of sheep and cattle [8,17,18,19,20]. Adding *Saccharomyces cerevisiae* culture to calves over 2.5 months old could improve feed digestibility, promote growth, and increase daily weight gain [21,22]. For fattening beef cattle, *Saccharomyces cerevisiae* culture is beneficial to improve feed digestibility and daily gain [23,24].

Most previous studies reported the effects of *Saccharomyces cerevisiae* on ruminant production, but these studies were rarely carried out on heat-stressed goats. Dietary supplementation with *Saccharomyces cerevisiae* or their mixture with *Clostridium butyricum* could ameliorate rumen fermentation and growth performance of heat-stressed goats [25]. Based on the results of our previous study, the effects of a low dose (less than ten times the dose compared with that of our previous study) of *Saccharomyces cerevisiae* on rumen fermentation and the growth performance of heat-stressed goats were investigated both in vitro and in vivo in this study. This study has the instructive significance of using *Saccharomyces cerevisiae* more economically to alleviate the adverse influences of heat stress on rumen fermentation and growth performance in intensive goat production.

## 2. Materials and Methods

### 2.1. Goats, Diet, and Management

This study was carried out from June to October, 2021 and was approved by the Animal Care and Use Committee of Jilin Agricultural Science and Technology University (Approval number: 20221011). The study was conducted on twelve (5.0 ± 1.0 months) female Macheng Black × Boer crossed goats weighing 17 to 19 kg. These goats were kept in a naturally ventilated house with individual feeding pens (1.20 × 1.50 m). Goats were fed on a diet with 1.20 kg/day of alfalfa and ground corn at a ratio of 2.1:1.0 on a dry matter (DM) basis. The goats were fed twice daily at 8:00 a.m. and 5:00 p.m. and had free access to water. The ingredients and nutritional levels of the diet are given in Table 1.

### 2.2. To Obtain the Model of Heat-Stressed Goats

Referring to Cai et al. [3], the modeling processes were divided into two periods, i.e., the control period and the heat-stress period (HS). In the control period, twelve goats were kept in a house with the environment temperature and relative humidity at 25.0 ± 2.5 °C and 62.1 ± 1.8%, respectively, for 15 days. Then, to obtain the heat stress goat model, the environment temperature and relative humidity of the house were increased to 33.3 ± 1.2 °C and 72.3 ± 2.3%, respectively, for 15 days. An air heater and a water sprinkler were used to control the temperature and humidity in the house. The temperature-humidity index (THI) was used as an environmental indicator to determine whether the goat was exposed to heat stress. The THI can be calculated as follows: THI = db °F − {(0.55 − 0.55 RH) (db °F − 58)}, where db °F is the dry bulb temperature in Fahrenheit and RH is the relative humidity (%) [26]. In the control and HS period, the THIs were 73.04 and 84.76, respectively. According to Marai et al. [27], in an environment with a THI greater than 82, the ruminants were subjected to heat stress.

### 2.3. Saccharomyces Cerevisiae Supplement Experiments

*Saccharomyces cerevisiae* live bacteria of 2.0 × 10^10^ CFU/g were obtained from Angel Yeast Co., Ltd. (Yichang, China), and the supplemented levels were 0‰ (HS1), 0.30‰ (SC1), 0.60‰ (SC2), and 1.20‰ (SC3) of the DM concentration in the basal diet for fermentation in vitro. Approximately 0.40 g of a ground feedstuff with different *Saccharomyces cerevisiae*, 32.0 mL of McDougall’s buffer [28], and 8.0 mL of rumen fluids were combined in a 100 mL flask. Then, the flask was flushed, sealed, and covered with CO_2_, rubber stoppers, and aluminum foil, respecitvely. Next, the flasks were subjected to shaking incubation at 125 rpm and 39 °C for 24 h. The flask of each *Saccharomyces cerevisiae* supplement level was prepared in triplicate.

Twelve heat-stressed goats were divided into four groups and assigned to a 4 × 4 Latin square experimental design. Each experimental cycle lasted for 21 days. The supplemented levels of *Saccharomyces cerevisiae* were 0‰ (HS2), 0.30‰ (SC1A), 0.60‰ (SC2A), and 1.20‰ (SC3A) of the DM concentration in the basal diet for feeding experiment. In the morning feeding on days 18 to 20 within each trial cycle, 5.0 g of an exogenous indicator (Cr_2_O_3_) was added to the diet to determine the feed digestibility. Goats were fed a basal diet for 21 days between experiment cycles to eliminate the influence of *Saccharomyces cerevisiae* on goats.

### 2.4. Sampling

Blood and rumen fluids were collected on the last day of the control and HS period. After 24 h fasting, blood samples were collected in the morning and taken from the jugular vein of twelve goats. The blood was immediately used to isolate peripheral blood lymphocytes to detect HSP70 gene expression levels. The remaining part of blood samples were centrifuged at 3000 rpm for 10 min to obtain serum and stored at −20 °C for further analysis. The rumen fluids were collected in the morning after four h of feeding and using a soft plastic stomach tube with a Jinteng GM-0.33A vacuum pump (Tianjin, China) from all the twelve goats. The rumen fluids were filtered by four layers of gauze to obtain the filtrate. The filtrate was immediately used for in vitro fermentation, and the remaining part was stored at −20 °C for further analysis. After 24 h incubation, these flasks were placed on ice for 15 min to stop the incubation, and the cultures were collected and stored at −20 °C for further analysis. Fecal samples were collected before morning and afternoon feeding from the rectum of all the twelve goats on days 12 to 14, and the fecal samples in the same period were pooled and stored at −20 °C for further analysis. In *Saccharomyces cerevisiae* feeding experiments, on the last day of each experimental cycle after 4 h, of feeding in the morning, the rumen fluids were collected 4 h after the morning feeding. The collection, pre-treatment, and storage methods of rumen fluids were consistent with that of the heat stress goat modeling experiment abovementioned. Before the morning and afternoon feedings on days 19 to 21 within each experimental cycle, fecal samples were collected from all goats and pooled in the same group. The rumen fluids and fecal samples were stored at −20 °C for further analysis.

### 2.5. Measurements

According to the instructions, a Solarbio Science & Technology kit (P5290, Beijing, China) was used to isolate the peripheral blood lymphocytes from the whole blood. The total RNA of the peripheral blood lymphocytes was extracted by using TRIzol^®^ Reagent (15596018, Life Technologies, Carlsbad, CA, USA) following the manufacturer’s instructions. A Revert Aid First Strand cDNA Synthesis kit (K1621, Thermo Fisher Scientific, Waltham, MA, USA) was then used for reverse transcription. Primers were referred to as Chaidanya et al. [4] and Cai et al. [25] and were synthesized by Sangon Biotech Co., Ltd. (Shanghai, China). The details of the gene-specific primer sequences are given in Table 2. SYBR RT-PCR Kit (e257, Bio-Rad, Hercules, CA, USA) in conjunction with an ABI. QuanStudio TM6 flex real-time fluorescent quantitative PCR system (Life Technologies, Carlsbad, CA, USA) were used for the RT-PCR conduction. The PCR reaction conditions were 94 °C for 3 min, 30 cycles of 94 °C for 30 s, 50 °C for 45 s, and 72 °C for 45 s, and a final extension at 72 °C for 10 min. Each sample was analyzed in triplicate, and the levels of relative expression were quantified using the 2-AACt method [29]. A cortisol assay kit (H094, Nanjing Jiancheng Bioengineering Institute, Nanjing, China) was used to measure the concentration of serum cortisol in goats following the manufacturer’s instructions.

The rumen pH values were measured immediately when incubation was stopped by a Thermo Scientific digital pH meter (Waltham, MA, USA). This parameter was measured immediately after the rumen fluid collection in the feeding experiment. The rumen fluid cultures in vitro experiment or rumen fluid in feeding experiment were centrifuged at 12,000× *g* at 4 °C for 15 min, and the supernatants were collected. As described by Maitisaiyidi et al. [30], the ammonia nitrogen (NH_3_-N) was measured using a NanoDrop8000 ultraviolet-visible spectrophotometer (Thermo Fisher Scientific, Waltham, MA, USA). As described by Yang et al. [31], volatile fatty acids (VFAs) were determined using gas chromatography. In brief, 0.20 mL of supernatant and 1.0 mL of 25% (*w*/*v*) metaphosphoric acid were mixed and centrifuged at 10,000 r/min for 10 min. Then, the liquid supernatant was injected into a 30 m × 0.53 mm × 1.00 μm Chrompack CP-Wax 52 fused silica column in a gas chromatograph equipped with a Model 2010 flame ionization detector (Shimazu, Kyoto, Japan). The amount of feedstuff given and surplus was recorded daily for each goat to calculate the DMI. The body weights were recorded at the start and end of each experimental cycle to calculate the ADG. As described by the #930.15 method in AOAC [32], the DM was measured both in feedstuff and feces, and the digestibility (%) of DM was calculated as (DM content in feedstuff − DM content in feces) ÷ DM content in feedstuff × 100. Neutral detergent fiber (NDF) and acid detergent fiber (ADF) were measured both in feedstuff and feces as described by Goering and Van Soest [33]. The digestibility (%) of these two parameters was calculated as (NDF or ADF content in feedstuff− NDF or ADF content in feces)/NDF or ADF content in feedstuff × 100.

### 2.6. Statistical Analysis

Data were analyzed using Prism (v8.0.2) (GraphPad Software Inc., San Diego, CA, USA). The paired *t*-tests were performed to reveal significantly different parameters of rumen fermentation and growth between the control and HS periods. To reveal significant differences of these parameters among the groups of different *Saccharomyces cerevisiae*-supplemented levels, two-way analysis of variance (ANOVA) tests followed by post hoc Dunn test for multiple pairwise comparisons were performed. *p* values of less than 0.05 were considered statistically significant.

## 3. Results

### 3.1. Obtaining Heat-Stressed Goats

**Figure 1:**
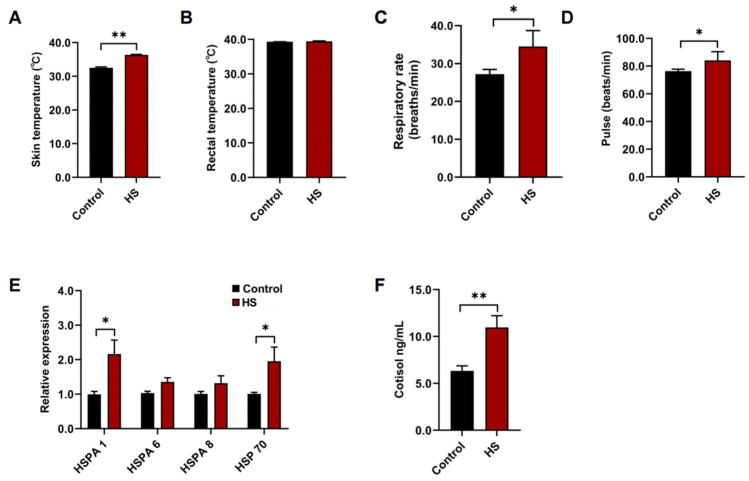
The parameters to evaluate the occurrence of heat stress in control and HS goats. The physiological parameters include (**A**) skin temperature, (**B**) rectal temperature, (**C**) respiratory rate, and (**D**) pulse rate of control and HS goats (*n* = 12). (**E**) The expression levels of HSP 70 family member genes in blood and rumen fluids (*n* = 6). (**F**) The cortisol concentrations in serum of control and HS goats. Data are expressed as the mean ± SEM * *p* < 0.05 and ** *p* < 0.01, indicating significant differences between the control and HS groups.

It was observed that the skin temperature, pulse, and respiratory rate were significantly increased (*p* < 0.05; Figure 1A,C,D), while there were no significant differences in the rectal temperatures (*p* > 0.05; Figure 1B) in HS goats compared with control goats. The expression levels of the heat shock protein 70 (HSP 70) family member genes were measured both in blood and rumen fluids. The expression levels of HSPA 1 in blood and the HSP 70 gene in rumen fluids were significantly increased (*p* < 0.05; Figure 1E), while there were no differences in the expression levels of HSPA 6 and HSPA 8 (*p* > 0.05; Figure 1E) in the blood lymphocytes in HS compared with control. Moreover, the serum cortisol concentration, which is an important index to evaluate occurrence of heat stress was significantly increased (*p* > 0.05; Figure 1F) in HS compared with control.

### 3.2. Heat Stress Adversely Influenced Rumen Fermentation Growth Performance of Goats

**Figure 2:**
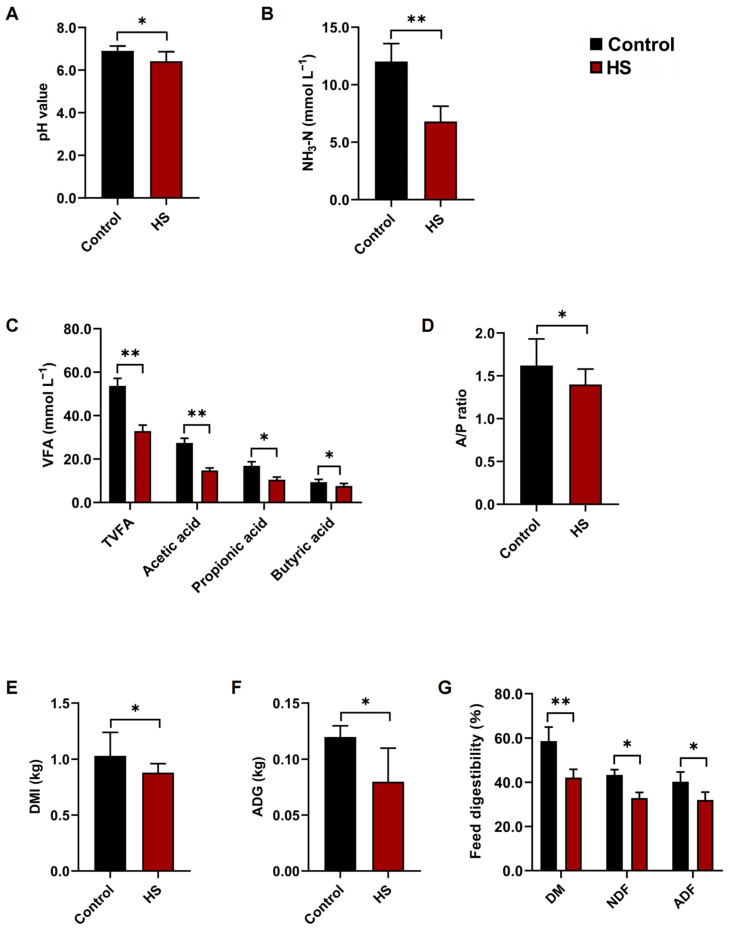
The rumen fermentation growth performance of control and HS goats. (**A**) The ruminal pH values of control and HS goats. The concentrations of (**B**) NH_3_-N, (**C**) total volatile fatty acid (TVFA), acetic acid, propionic acid, butyric acid, and (**D**) acetic acid to propionic acid ratios (A/P ratios) in the rumen of control and HS goats. (**E**) The dry matter intake (DMI), (**F**) the average daily gain (ADG), and (**G**) the digestibilities of dry matter (DM), neutral detergent fiber (NDF), and acid detergent fiber (ADF) of control and HS goats. Data are expressed as the means ± SEM. * *p* < 0.05 and ** *p* < 0.01, indicating significant differences between the control and heat stress periods.

It was observed that the HS exhibited significantly lower pH values (*p* < 0.05; Figure 2A), the concentrations of NH_3_-N (*p* < 0.01; Figure 2B), total volatile fatty acid, acetic acid, propionic acid, and butyric acid, and acetic acid to propionic acid ratios (A/P ratios) than that exhibited in the control group. Moreover, the HS showed significantly lower DMI, ADG, and the digestibilities of DM, NDF, and ADF (*p* < 0.05; Figure 2E–G) than that of the control group.

### 3.3. Saccharomyces Cerevisiae Improves Rumen Fermentation and Feeds Digestibility In Vitro

**Figure 3:**
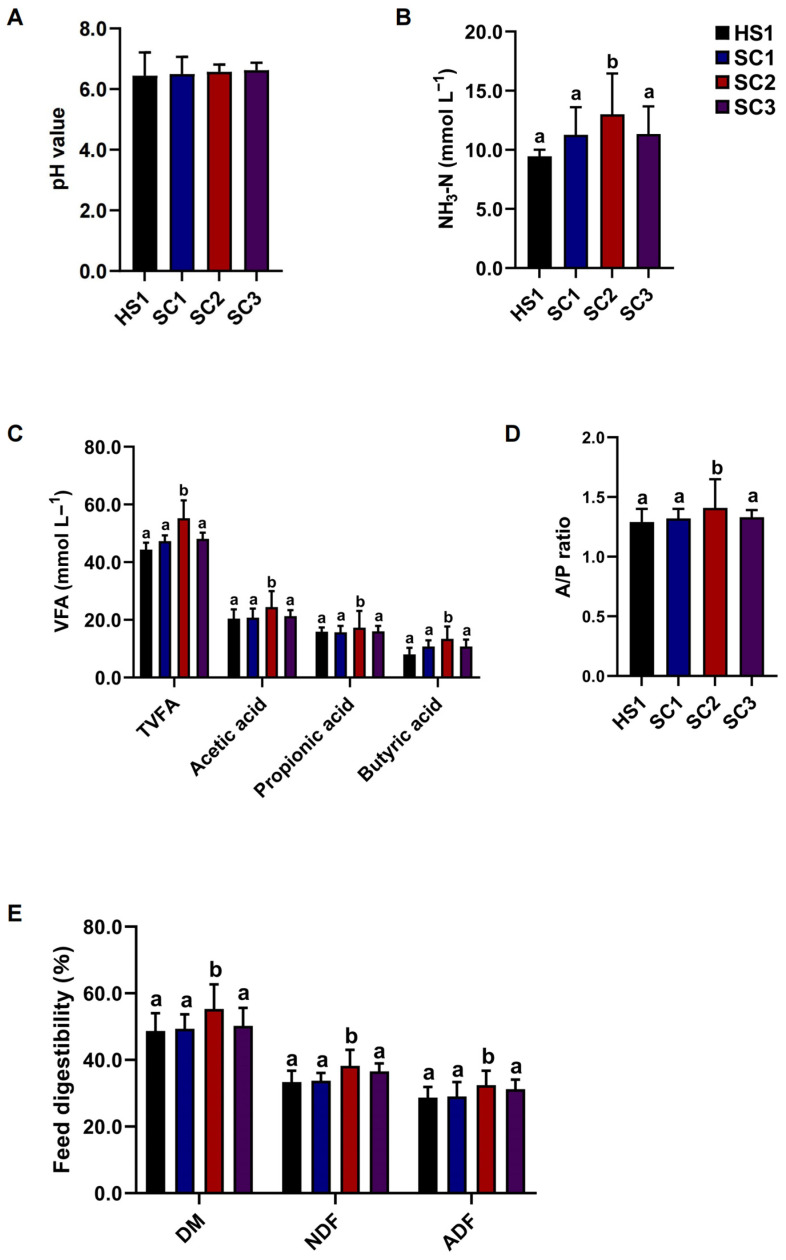
Fermentation parameters with *Saccharomyces cerevisiae* supplementation in vitro. The (**A**) pH values, (**B**) NH_3_-N concentrations, (**C**) VFA, including acetic acid, propionic acid, and butyric acid, and (**D**) A/P ratios in ruminal cultures of HS1, SC1, SC2, and SC3. (**E**) The feed digestibilities, including the digestibilities of DM, NDF, and ADF in rumen fluid cultures of HS1, SC1, SC2, and SC3. The different lowercase superscripts in the rows indicate significant differences (*p* < 0.05); the same lowercase superscripts in the rows indicate no differences (*p* > 0.05).

In the fermentation experiment, there were no significant differences in ruminal pH values among HS1, SC1, SC2, and SC3 (*p* > 0.05; Figure 3A). However, the concentrations of NH_3_-N, TVFA, acetic acid, and propionic acid, and the A/P ratio in ruminal cultures were significantly increased (*p* < 0.05; Figure 3B–D) in SC2 compared with the HS1, SC1, and SC2, while there were no significant differences among HS1, SC1, and SC3 (*p* > 0.05; Figure 3B–D). Moreover, the digestibilities of DM, NDF, and ADF were significantly increased (*p* < 0.05; Figure 3E) in SC2 compared with HS1, SC1, and SC3, while there were no significant differences among HS1, SC1, and SC3 (*p* > 0.05; Figure 3E).

### 3.4. Saccharomyces Cerevisiae Improves Rumen Fermentation and Growth Performance of Heat-Stressed Goats

**Figure 4:**
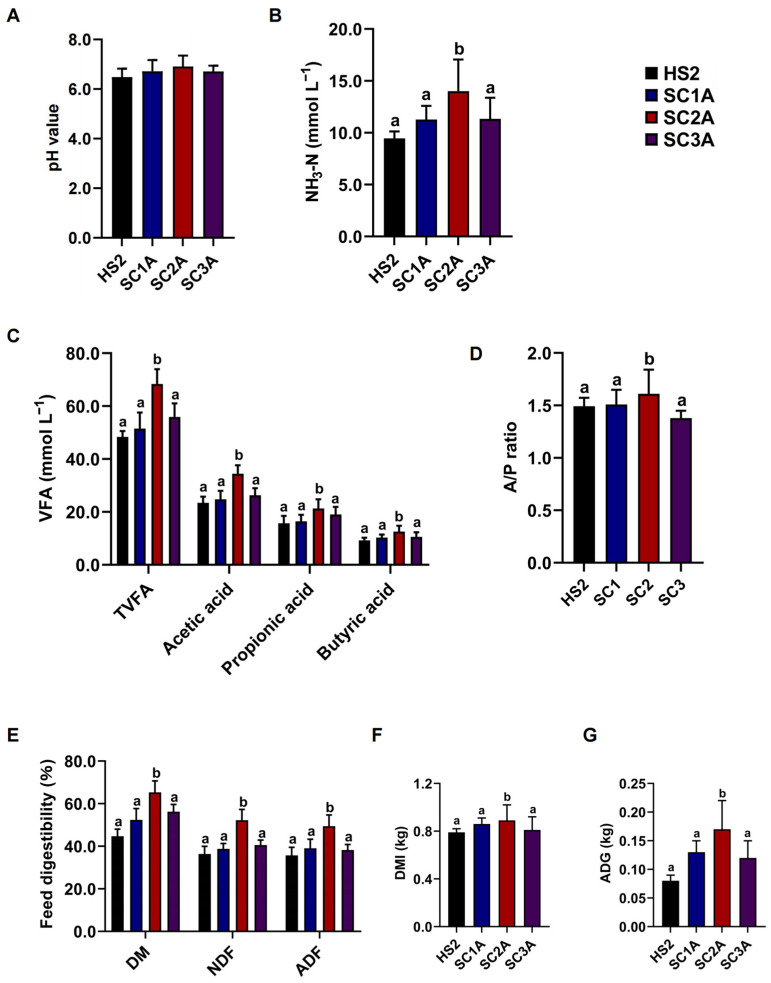
Fermentation parameters with *Saccharomyces cerevisiae* supplementation in vivo. The (**A**) pH values, (**B**) NH_3_-N concentrations, (**C**) VFA, including acetic acid, propionic acid, and butyric acid, and (**D**) A/P ratios in ruminal cultures of HS2, SC1A, SC2A, and SC3A. (**E**) The DMI, (**F**) ADG, and (**G**) the feed digestibilities of DM, NDF, and ADF in rumen fluids of HS2, SC1A, SC2A, and SC3A. The different lowercase superscripts in the rows indicate significant differences (*p* < 0.05); the same lowercase superscripts in the rows indicate no differences (*p* > 0.05).

To investigate the effects of *Saccharomyces cerevisiae* on rumen fermentation by feeding experiments, we found that the ruminal pH, the concentrations of NH_3_-N, TVFA, acetic acid, and propionic acid, and the A/P ratio were significantly increased (*p* < 0.05; Figure 4A–D) in the SC2A group compared with HS2, SC1A, and SC3A, while there were no significant differences among HS2, SC1A, and SC3A (*p* > 0.05; Figure 4A–D). To investigate the effects of *Saccharomyces cerevisiae* on the feed digestibilities by feeding trials, we found that the digestibilities of DM, NDF, and ADF were significantly increased (*p* < 0.05; Figure 4E) in SC2A compared with HS2, SC1A, and SC3A, while there were no significant differences among HS2, SC1A, and SC3A (*p* > 0.05; Figure 4E).

## 4. Discussion

Heat stress is inevitable in intensive goat production during hot summer [1,3,25]. The occurrence of heat stress in goats was judged accurately is the guarantee of the follow-up experiment of this study. Previous studies have reported several methods to assess the occurrence of heat stress in goats: monitoring the environmental temperature and relative humidity, measuring the physiological parameters (skin temperature, rectal temperature, pulse, and respiratory rate), determining the expression levels of HSP 70 genes in blood and rumen fluid, and measurement the cortisol concentrations in serum [3,4,25]. However, only one indicator of these indicators is used to determine whether heat stress has occurred in a single study. This evaluation of the occurrence of heat stress may be inaccurate. Therefore, we combined several existing evaluation indicators to confirm the actual occurrence of heat stress. On this basis, a follow-up experiment was carried out. Previous studies have reported that heat stress could cause adverse influences on rumen fermentation by decreasing ruminal pH values, the concentrations of NH_3_-N, TVFA, acetic acid, propionic acid, butyric acid, and A/P ratios [3,34,35,36,37]. The results of these rumen fermentation parameters in this study were consistent with previous studies. The adverse effects of heat stress on rumen fermentation were demonstrated again in goats. These results further lay a foundation for the further experiment in this study.

Dietary supplementation with *Saccharomyces cerevisiae* is one effective way to enhance rumen fermentation and growth performance of ruminants. In this study, we found that 0.6‰ *Saccharomyces cerevisiae* supplement level had the same effects on the parameter of rumen fermentation and growth performance compared to the 0.6% *Saccharomyces cerevisiae* supplement level in our previous study. In the present study, the ruminal pH increased with *Saccharomyces cerevisiae* supplementation in the feeding experiment. This is consistent with the previous study on cows with live yeast supplementation in the hot season [38]. These results suggested that *Saccharomyces cerevisiae* could alleviate the decreased pH from heat stress [1]. This increasing effect may be attributed to this probiotic potentially creating a more O_2_-free ruminal environment that is beneficial for enhancing the relative abundance of lactate-utilizing bacteria and improving lactate consumption in the rumen; therefore, the rumen pH was stabilized [10,11,12,13]. In contrast, some studies reported that supplementation with *Saccharomyces cerevisiae* had no effect on the ruminal pH or could decrease it [15,39,40]. The differences could be attributed to the different sources, strains, and doses of this probiotic and livestock in different physiological stages and housing environments. In this study, supplementation with *Saccharomyces cerevisiae* could increase ruminal NH_3_-N concentration. This result is inconsistent with previous studies suggesting that yeast supplementation led to a significant decrease or did not affect the NH_3_-N concentration in the rumen [11,15,16,41]. The increase in the NH_3_-N concentration could be attributed to the *Saccharomyces cerevisiae* facilitating the relative abundance of rumen microbiota to degrade feed protein [15]. In this study, the ruminal TVFA concentration was increased in heat-stressed goats. This result is consistent with previous studies that suggested supplementing dry yeast or *Saccharomyces cerevisiae* ruminants caused a significant increase in the rumen of heat-stressed goats [8,17,18,19,20,25,42]. VFA in the rumen originates from the fermentation of fiber in feedstuff; *Saccharomyces cerevisiae* facilitate the growth and reproduction of cellulolytic bacteria in the rumen [10,11], enhancing the VFA production in the rumen. In this study, the DMI of heat-stressed goats was increased with 0.6‰ *Saccharomyces cerevisiae* supplementation. This result is similar to our previous study, which showed that supplementation with 0.60% *Saccharomyces cerevisiae* increased the DMI in the heat-stressed goats [25]. However, another study showed that supplementation with *Saccharomyces cerevisiae* did not affect the DMI of cows under heat stress conditions [43]. Besides, our study showed that *Saccharomyces cerevisiae* improved the ADG of heat-stressed goats with 0.6‰ *Saccharomyces cerevisiae* supplementation, similar to the results of heat-stressed goats with 0.6% *Saccharomyces cerevisiae* supplementation [25]. In this study, *Saccharomyces* *cerevisiae* supplementation improved the digestibilities of DM, NDF, and ADF. This result is consistent with previous studies suggesting that the digestibilities of DM, NDF, and ADF were improved by supplementation with *Saccharomyces cerevisiae* in goats and sheep [25,40,44]. This result may be attributed to yeast cells containing glucose, furan mannose, and chitin, which can be used as fermentation substrates for rumen microbiota to promote their activities and digestibility. [45,46]. Future studies to investigate the effects of *Saccharomyces cerevisiae* on the rumen microbiota of heat-stressed goats could lead to a better understanding of their contribution to rumen fermentation and their growth parameters.

## 5. Conclusions

The low dose of *Saccharomyces cerevisiae* could ameliorate rumen fermentation by elevating ruminal pH, increasing NH_3_-N and VFA concentrations, and enhancing feed digestibility, resulting in promoted growth performance in increasing the ADG of heat-stressed goats. In summary, dietary supplementation with *Saccharomyces cerevisiae* can effectively alleviate the adverse effects of heat stress on rumen fermentation and the growth performance of goats. For this purpose, the appropriate supplement level of this probiotic is 0.60‰ of the dry matter concentration in the basal diet. This study provides a reference for the effective and more economical application of *Saccharomyces cerevisiae* in the future hot seasons.

## Figures and Tables

**Table 1:** The ingredients and nutritional levels (g/kg) in the diet of goats.

Ingredient	Content	Nutrition Level	Amount
Alfalfa	560	Dry matter	954
Ground corn	266	Organic matter	851
Soybean meal	80	Crude protein	176
Wheat barn	77	Neutral detergent fibre	435
Ca_2_HPO_4_	7	Acid detergent fiber	261
Premix *	10	Ca	5.9
		P	3.2

* Premix contained per kg: 20.70 g Mg, 0.50 g Fe, 1 g Mn, 2 g Zn, 43 mg Se, 47 mg I, 54 mg, Co, 90,000 IU vitamin A, 17,000 IU vitamin D, 1750 IU vitamin E.

**Table 2:** The primer sequences of HSP 70 genes.

Gene	Primer Sequence	Product Length	Annealing Temperature	GenBank Accession No.
B-actin	F: TCTGGCACCACACCTTCTAC R: TCTTCTCACGGTTGGGCCTTG	102	60	XM 018039831.1
HSPA 1	F: CGACCAGGGAAACCGGCAC R: CGGGTCGCCGAACTTGC	151	60	NM 005677146.3
HSPA 6	F: TCTGCCGCAACAGGATAAA R: CGCCCACGCACGAGTAC	239	60	NM_001314233.1
HSPA 8	F: ACCTCTATTACCCGTGCCC R: CTCTTATTCAGTTCCTTCCCATT	203	60	XM 018039831.1
HSP 70	F: TGGCTTTCACCGATACCGAG R: GTCGTTGATCACGCGGAAAG	167	60	NM 001285703.1

## Data Availability

Not applicable.

## References

[1] Cai L.Y., Yu J.K., Zhang J., Qi D.S. (2015). The effects of slatted floors and manure scraper systems on the concentrations and emission rates of ammonia, methane and carbon dioxide in goat buildings. Small Rumin. Res..

[2] Zhao Y.Z. (2011). Goat and sheep production. Chapter VIII: Feeding and Management.

[3] Cai L.Y., Yu J.K., Hartanto R., Zhang J., Yang A., Qi D.S. (2019). Effects of heat challenge on growth performance, ruminal, blood and physiological parameters of Chinese crossbred goats. Small Rumin. Res..

[4] Chaidanya K., Soren N.M., Sejian V., Bagath M., Manjunathareddy G.B., Kurien E.K., Varma G., Bhatta R. (2017). Impact of heat stress, nutritional stress and combined (heat and nutritional) stresses on rumen associated fermentation characteristics, histopathology and HSP70 gene expression in goats. J. Anim. Behav. Biometeorol..

[5] Zhao S.G., Li M., Zheng N., Wang J.Q. (2019). Effect of heat stress on bacterial composition and metabolism in the rumen of lactating dairy cows. Animals.

[6] He Y.Q., Li Y.Q., Yang Y.K., Liu X.Y., Liu G.B., Sun B.L., Liu D.W. (2018). Research Advances on Heat Stress in Goats. Chin. Anim. Husb. Vet. Med..

[7] Hill C., Guarner F., Reid G., Gibson G.R., Merenstein D.J., Pot B., Morelli L., Canani R.B., Flint H.J., Salminen S. (2014). The international scientific association for probiotics and prebiotics consensus statement on the scope and appropriate use of the term probiotic. Nat. Rev. Gastroenterol. Hepatol..

[8] Desnoyers M., Reverdin S.G., Bertin G., Pouter C.D., Sauvant D. (2009). Meta-analysis of the influence of *Saccharomyces cerevisiae* supplementation on ruminal parameters and mile productuin of ruminant. J. Dairy Sci..

[9] Nie L., Zhang A.Z., Jiang N., Yang Z.N. (2017). Application of probiotics in the production of juvenile ruminants. Feed. Rev..

[10] Chaucheyras-Durand F., Fonty G. (2002). Influence of a probiotic yeast (*Saccharomyces cerevisiae* CNCM I-1077) on microbial colonization and fermentations in the rumen of newborn lambs. Microbial. Eco. Health Dis..

[11] Chaucheyras-Durand F., Walker N.D., Bach A. (2008). Effects of active dry yeasts on the rumen microbial ecosystem: Past, present and future. Anim. Feed Sci. Tech..

[12] Paul R.B., Jeffery A.C., Nicole C.B.S. (2015). Live yeast and yeast cell wall supplements enhance immune function and performance in food-producing livestock: A review. Microorganisms.

[13] Reis L.F., Sousa R.S., Oliveira F.L.C., Rodrigues F.A.M.L., Araújo C.A.S.C., Meira-Júnior E.B.S. (2018). Comparative assessment of probiotics and monensin in the prophylaxis of acute ruminal lactic acidosis in sheep. BMC Vet. Res..

[14] Dias A.L.G., Freitas J.A., Micai B., Azevedo R.A., Greco L.F., Santos J.E.P. (2017). Effect of supplemental yeast culture and dietary starch content on rumen fermentation and digestion in dairy cows. J. Dairy Sci..

[15] Oeztuerk H. (2009). Effects of live and autoclaved yeast cultures on ruminal fermentation in vitro. J. Anim. Feed Sci..

[16] Kiran R.R., Kumar D.S. (2013). Influence of yeast culture supplementation on rumen fermentation of bulls fed complete rations. IJASVM.

[17] Schingoethe D.J., Linke K.N., Kalscheur K.F., Hippen A.R., Rennich D.R., Yoon I. (2004). Feed efciency of mid-lactation dairy cows fed yeast culture during summer. J. Dairy Sci..

[18] Křižova L., Richter M., Třinacty J., Řiha J., Kumprechtová D. (2011). The effect of feeding live yeast cultures on ruminal pH and redox potential in dry cows as continuously measured by a new wireless device. Czech. J. Anim. Sci..

[19] El-Waziry A.M., Ibrahim H.R. Effect of *Saccharomyces cerevisiae* on cell wall constituents digestion in sheep fed berseem (*Trifolium alexandrinum*) hay and cellulase activity. Proceedings of the International Conference on the Arabian Oryx in the Arabian Peninsula.

[20] Patra A.K. (2012). The use of live yeast products as microbial feed additives in ruminant nutrition. Asian J. Anim. Vet. Adv..

[21] Guo Y.Q., Zhao Y.F., Zhang X.Y. (2019). Effect of yeast culture on growth performance and rumen fermentation of weaned calves. Feed Res..

[22] Zhu S.Z., Zhao G.K., Xie J.L., Zhang J.Q., Yang B.H., Wang W.P., Yu H.Y., Dong B., Ma Y.P., Zhang G.P. (2019). Effect of adding yeast culture ayc-x6 to calf diet on growth and development in calves. Chin. Cattle Sci..

[23] Wagner J.J., Engle T.E., Belknap C.R., Dorton K.L. (2016). Meta-analysis examining the effects of *Saccharomyces cerevisiae* fermentation products on feedlot performance and carcass traits. Pro. Anim. Sci..

[24] Huang W.M., Tan L., Wang F., Kang L., Li X.B., Zuo F.Y. (2019). Effects of yeast culture on growth performance, slaughter performance, and meat quality of finishing cattle. Chin. J. Anim. Nutr..

[25] Cai L.Y., Yu J.K., Hartanto R., Qi D.S. (2021). Dietary supplementation with *Saccharomyces cerevisiae*, *Clostridium butyricum* and their combination ameliorate rumen fermentation and growth performance of heat-stressed goats. Animals.

[26] LPHSI (1990). Livestock and Poultry Heat Stress Indices Agriculture Engineering Technology Guide.

[27] Marai I.F.M., EI-Darawany A.A., Fadie A., Abdel-Hafez M.A.M. (2007). Physiological traits as affected by heat stress in sheep—A review. Small Rumin. Res..

[28] McDougall E.I. (1948). Studies on ruminant saliva. 1 The composition and output of sheep’s saliva. Biochem. J..

[29] Ramakersm C., Ruijter J.M., Deprez R.H., Moorman A.F. (2003). Assumption-free analysis of quantitative real-time polymerase chain reaction (PCR) data. Neurosci. Lett..

[30] Maitisaiyidi T., Yibureyimu A., Yang K. (2012). Determination of ammonia-nitrogen in ruminal fluid treated with methanol by alkaline hypochlorite-phenol spectrophotometry. Xinjiang Agr. Sci..

[31] Yang W.Z., Beauchemin K.A., Rode L.M. (2001). Effects of grain processing, forage to concentrate ratio, and forage particle size on rumen pH and digestion by dairy cows. J. Dairy Sci..

[32] AOAC (2005). Official Methods of Analysis.

[33] Goering H.K., Van Soest P.J. (1970). Forage fiber analysis (apparatus, reagents, procedures and some applications). USDA Agriculture Handbook.

[34] Tajima K., Nonaka I., Higuchi K., Takusari N., Kurihara M., Takenak A., Mitsumori M., Kajikawa H., Aminov R.I. (2007). Influence of high temperature and humidity on rumen bacterial diversity in Holstein heifers. Anaerobe.

[35] Nonaka I., Takusari N., Tajima K., Suzuki T., Higuchi K., Kurihara M. (2008). Effects of high environmental temperatures on physiological and nutritional status of prepubertal Holstein heifers. Livest. Sci..

[36] Uyeno Y., Sekiguchi Y., Tajima K., Takenaka A., Kurihara M., Kamagata Y. (2010). An rRNA-based analysis for evaluating the effect of heat stress on the rumen microbial composition of Holstein heifers. Anaerobe.

[37] Yadav B. (2013). Impact of heat stress on rumen functions. Vet. World.

[38] Moallem I.U., Lehrer H., Livshitz L., Zachut M., Yakoby S. (2009). The effects of live yeast supplementation to dairy cows during the hot season on production, feed efficiency, and digestibility. J. Dairy Sci..

[39] Lascano G.J., Zanton G.I., Heinrichs A.J. (2009). Concentrate levels and *Saccharomyces cerevisiae* affect rumen fluid-associated bacteria numbers in dairy heifers. Livest. Sci..

[40] Hossain S.A., Parnerkar S., Haque N., Gupta R.S., Kumar D., Tyagi A.K. (2012). Influence of dietary supplementation of live yeast (*Saccharomyces cerevisiae*) on nutrient utilization, ruminal and biochemical profiles of Kankrej calves. Int. J. Appl. Anim. Sci..

[41] Thrune M., Bach A., Ruiz-Moreno M., Stern M.D., Linn J.G. (2009). Effects of *Saccharomyces cerevisiae* on ruminal pH and microbial fermentation in dairy cows: Yeast supplementation on rumen fermentation. Livest. Sci..

[42] Ondarza M.B., Sniffen C.J., Dussert L., Chevaux E., Sullivan J., Walker N. (2010). CASE STUDY: Multiple-study analysis of the effect of live yeast on milk yield, milk component content and yield, and feed efficiency. Prof. Anim. Sci..

[43] Bach A., Iglesias C., Devant M. (2007). Daily ruminal pH pattern of loose-housed dairy cattle as affected by feeding pattern and live yeast supplementation. Anim. Feed Sci. Tech..

[44] Lila Z.A., Mohammed N., Yasui T., Kurokawa Y., Kanda S., Itabashi H. (2004). Effects of a twin strain of *Saccharomyces cerevisiae* live cells on mixed ruminal microorganism fermentation in vitro. J. Anim. Sci..

[45] Klis F.M. (1994). Review: Cell wall assembly in yeast. Yeast.

[46] Moukadiri I., Armero J., Abad A., Sentandreu R., Zueco R. (1997). Identification of a mannoprotein present in the inner layer of the cell wall of *Saccharomyces cerevisiae*. J. Bacteriol..

